# Comparative analysis of molecular and chromatographic methods for detecting palm oil adulteration in yogurt

**DOI:** 10.1038/s41598-025-15523-6

**Published:** 2025-08-12

**Authors:** Mohammad Dowlatabadi, Seyed Ali Mortazavi, Hasan Ravansalar, Mohammad Reza Saeidi Asl, Ahmad Pedram Nia

**Affiliations:** 1 Department of Food Science and Technology, Sab.C., Islamic Azad University, Sabzevar, Iran; 2 Department of Food Science and Technology, Faculty of Agriculture, Ferdowsi University of Mashhad, Mashhad, Iran; 3 Department of Microbiology, Faculty of Medicine, Sabzevar University of Medical Science, Sabzevar, Iran

**Keywords:** Gas chromatography (GC-FID), MT3-B gene, Palm oil adulteration, Phytosterol profiling, Quantitative PCR (qPCR), Yogurt fat, Molecular biology, Analytical chemistry

## Abstract

Palm oil adulteration in dairy products poses significant concerns for food authenticity, consumer protection, and regulatory compliance. This study presents a validated combined molecular and chromatographic detection strategy to detect and quantify palm oil adulteration in yogurt fat. Yogurt fat samples were fortified with palm olein at concentrations ranging from 1 to 100% (w/w). DNA was extracted using a modified CTAB protocol and analysed for the oil palm-specific MT3-B gene via conventional and quantitative PCR (qPCR). Concurrently, Gas Chromatography–Flame Ionization Detection (GC-FID) was used to quantify phytosterols in the unsaponifiable lipid fraction. To address this issue, we developed a dual-analytical strategy combining qPCR targeting the chloroplast MT3-B gene of oil palm (Elaeis guineensis) and gas chromatography for sterol profiling. The qPCR assay demonstrated high specificity and sensitivity, with a detection limit of 0.01 ng and a quantification limit of 0.02 ng. GC-FID analysis showed a dose-dependent increase in phytosterol content, with pronounced rises at 50% and 100% substitution. A strong correlation (r = 0.89) was observed between qPCR-detected DNA levels and GC-measured phytosterol concentrations. To evaluate real-world applicability, the method was applied to 15 commercial yogurt samples, all of which tested positive for molecular and chemical markers of palm oil. This integrated approach offers a rapid and reliable strategy for detecting palm oil adulteration, supporting food quality assurance and regulatory enforcement.

## Introduction

The adulteration of dairy products, particularly yogurt, with lower-cost vegetable oils such as palm oil has emerged as a pressing concern for food authenticity, consumer protection, and public health. Economic motivations often drive the substitution of milk fat with plant oils, distorting the nutritional profile of dairy foods while simultaneously introducing risks associated with oil processing contaminants such as 3-MCPD and glycidyl esters^1,2^. Regulatory bodies, including the U.S. Food and Drug Administration, have identified economically motivated adulteration as a critical food safety issue and emphasised the importance of validated detection methods^3^. Beyond regulatory compliance, growing evidence suggests that adulterated dairy products may contribute to adverse health outcomes, particularly cardiovascular risks associated with altered lipid compositions^4^.

To date, there is limited scientific literature documenting direct adulteration of yogurt with palm oil. However, palm-derived lipids—including monoacylglycerols and palm stearin—are commonly used in dairy analogues, processed cheese, and yogurt stabilizers, often without explicit disclosure on product labelling. The lack of transparency surrounding these additives has raised concerns in food fraud investigations and regulatory surveillance. Given the economic incentive to substitute milk fat with lower-cost vegetable oils, and the absence of standardized detection protocols in fermented dairy matrices, the development of robust analytical tools for verifying fat composition in yogurt is both relevant and necessary. While direct evidence of palm oil adulteration in yogurt remains limited, several studies have documented the use of non-dairy fats in milk and cheese products as an economically motivated adulteration strategy. Ntakatsane et al. (2013) demonstrated that triacylglycerol profiling can differentiate milk fat from vegetable oils in dairy matrices such as cheese and cream^5^. Similar analytical challenges apply to yogurt, where emulsified plant lipids may be introduced either as direct adulterants or as functional additives. While robust, these methods often require laborious sample preparation and are not always suited for high-throughput environments^6^.

Emerging alternatives, such as fluorescence spectroscopy combined with chemometric modelling, have demonstrated potential for rapid adulteration detection in edible oil systems^7^. Recent advances in laboratory automation (e.g., robotic pipetting, automated lipid extraction) and miniaturized platforms (e.g., microfluidic systems) offer promising avenues for reducing manual workload and enhancing reproducibility in routine quality control settings.

The novelty of this study lies in the combined use of molecular and chromatographic techniques—specifically, qPCR targeting the MT3-B gene and GC-FID sterol profiling—to detect palm oil adulteration in yogurt fat. Yogurt presents an analytically challenging matrix due to its emulsified fat and protein content, which can interfere with both DNA extraction and chemical quantification. While both qPCR and GC-based sterol analysis have been applied independently in food fraud detection, their joint application in a dairy context offers complementary strengths: species-specific confirmation via DNA and quantitative profiling of plant oil markers via sterols.

Traditionally, the detection of non-milk fats in dairy matrices has relied on chemical profiling. Chromatographic techniques—especially gas chromatography coupled with flame ionisation detection (GC-FID) or mass spectrometry (GC–MS)—are widely employed to detect phytosterols, which serve as biomarkers indicative of plant oil adulteration^8,9^. While robust, these methods often require laborious sample preparation and are not always suited for high-throughput environments^6^.

Molecular techniques have increasingly been adopted to complement chemical analysis by enabling species-specific identification of plant-based adulterants. DNA-based assays, such as quantitative PCR (qPCR), can detect trace amounts of DNA from oil palm (Elaeis guineensis) even in emulsified or thermally processed products, offering a sensitive and specific detection option.

Although extensive research has addressed edible oil adulteration in general, limited evidence exists regarding palm oil adulteration in yogurt specifically. Prior investigations have largely focused on milk or cheese matrices, and reports of undeclared palm oil in yogurt remain scarce. This gap presents a challenge for regulators and food quality professionals seeking to protect consumers from non-dairy fat inclusion in cultured dairy products.

This study evaluates and applies a combined molecular and chromatographic detection strategy integrating MT3-B gene-based qPCR and GC-FID phytosterol profiling to detect palm oil adulteration in yogurt fat. In addition to controlled spiking experiments, this approach was applied to the analysis of 15 commercial yogurt samples to assess its performance under realistic conditions. By pairing DNA-based identification with sterol profiling, the method aims to deliver a sensitive, confirmatory, and adaptable framework for routine food authenticity enforcement in dairy systems. In this study, the chloroplast-encoded MT3-B gene was selected as a molecular marker for palm oil detection. This gene is specific to Elaeis guineensis and has been shown to persist in processed food matrices. Prior studies have confirmed its utility for species-specific detection due to its high copy number and low sequence homology with other oilseed plants^10^.This assay enables the identification of trace DNA from palm-derived ingredients, even in emulsified or thermally processed dairy products.

## Results

### MT3-B gene detection

#### UV spectroscopy as preliminary screening

**Fig. 1:**
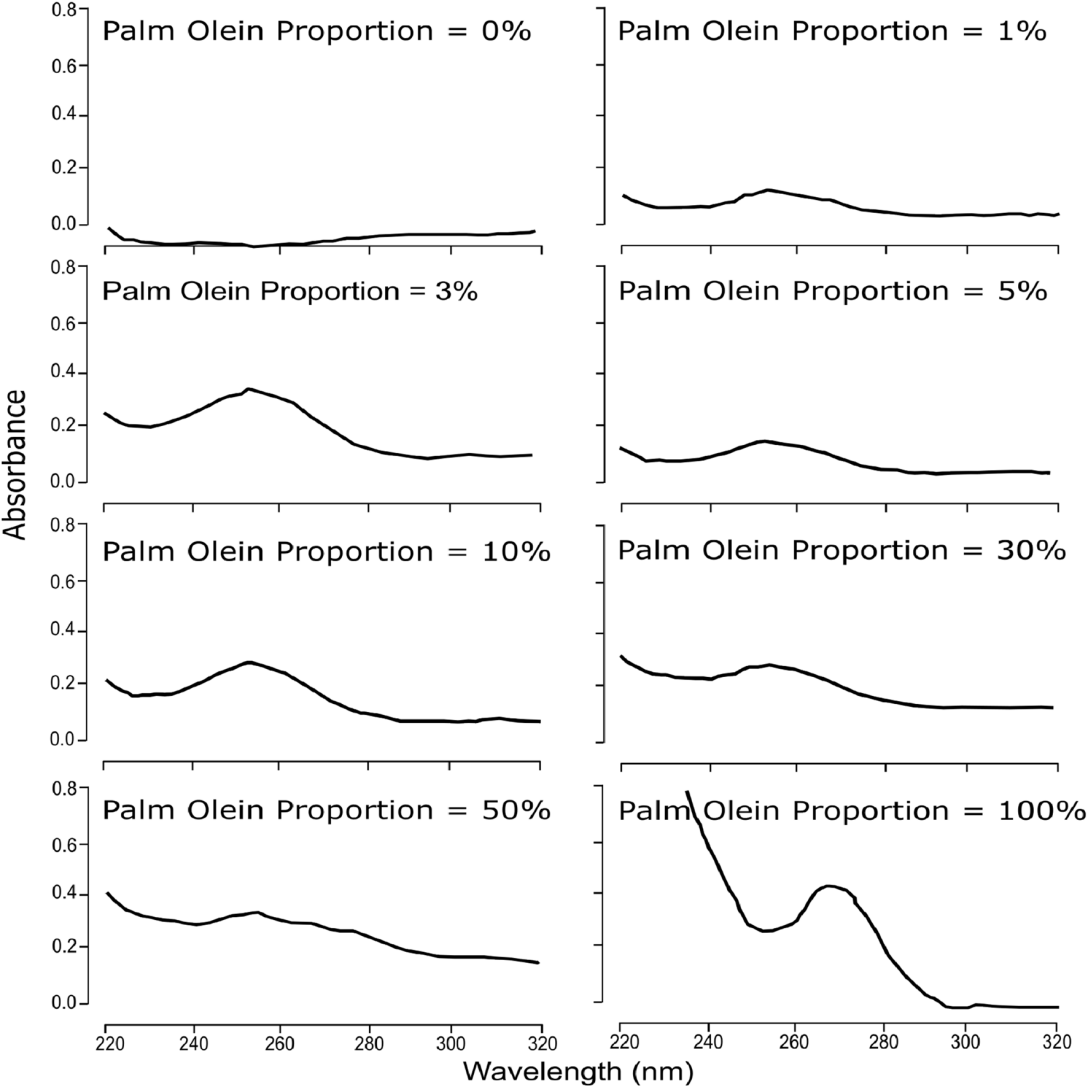
UV absorption spectra of yogurt fat samples containing 0–100% palm olein. The 100% sample is retained only as a qualitative reference to demonstrate full matrix shift and is not used in regression analysis.

Although UV absorbance generally increased with palm olein concentration, the trend was not strictly linear between 10 and 100% substitution (Fig. 1). This non-linearity likely arises from matrix-dependent effects such as spectral overlap from secondary oxidation products, saturation of chromophoric moieties, and interactions among lipid species in the emulsified system. Similar deviations from linearity in absorbance behaviour have been reported for edible oils undergoing thermal or compositional modifications^9^. These factors should be considered when interpreting UV absorbance as a semi-quantitative indicator of adulteration. Although a general increase in UV absorbance was observed with higher palm olein concentrations (Fig. 1), the relationship was not strictly linear. This non-linearity likely arises from matrix-related effects such as spectral overlap, high chromophore concentration leading to absorbance saturation, and interference from lipid oxidation products present in dairy fat.These factors are known to influence UV absorption in complex food systems and limit its specificity for quantitative detection^9^. Therefore, we interpret UV spectroscopy as a rapid, semi-quantitative screening method rather than a standalone diagnostic tool.

Ultraviolet (UV) absorbance spectra of yogurt fat samples fortified with 0–50% palm olein showed weak and non-linear trends across the 220–320 nm range. Although some increase in absorbance was observed at higher adulteration levels, overlapping spectral features from milk fat and plant sterols hindered quantitative interpretation. These results confirm that UV spectroscopy, while fast and instrumentally accessible, lacks the specificity and linear response needed for reliable quantification. However, its rapidity may still make it useful for preliminary qualitative screening of gross adulteration in processed dairy products.

#### DNA yield and gel electrophoresis

**Fig. 2:**
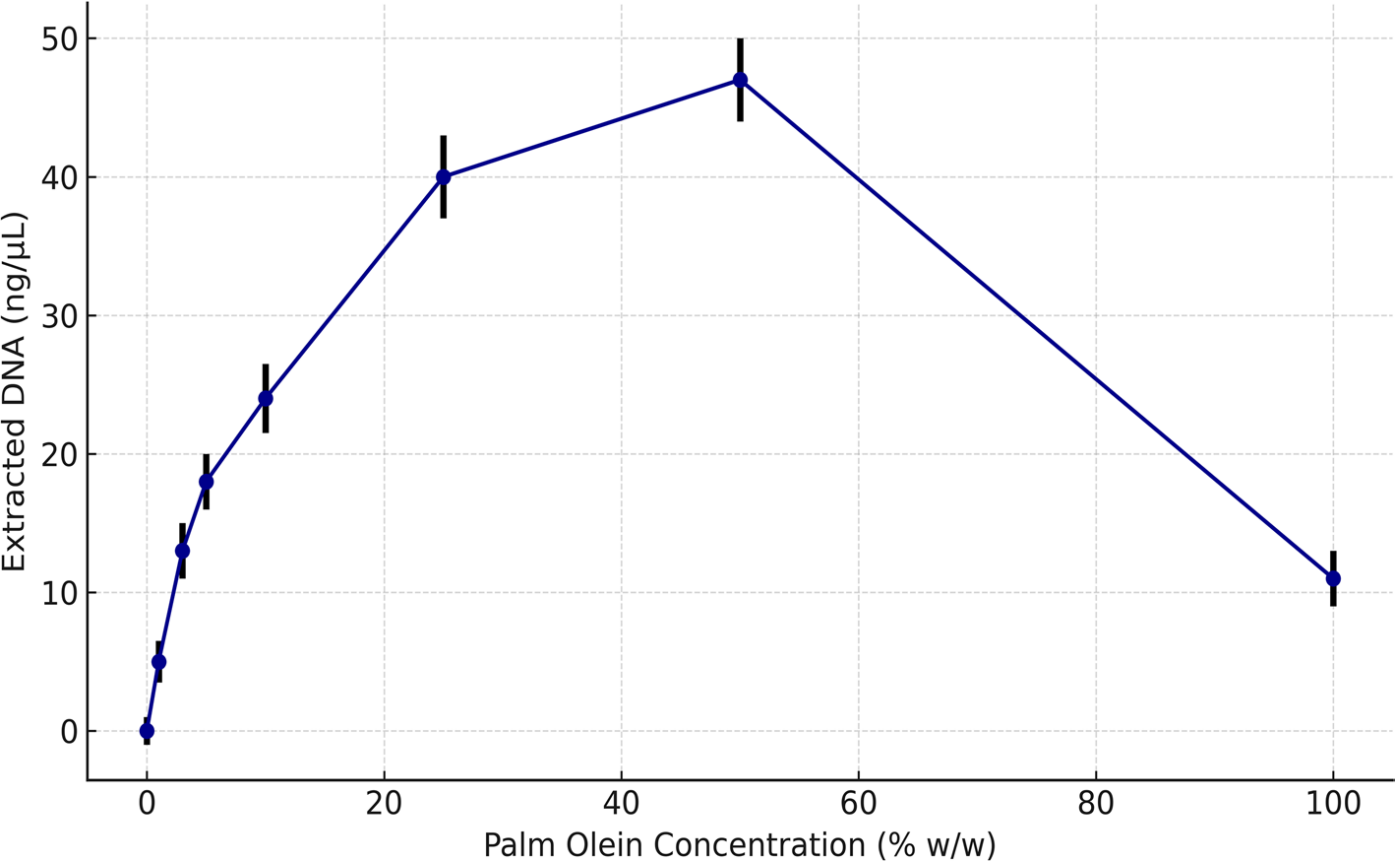
Extracted DNA yield from yogurt fat samples adulterated with varying concentrations of palm olein. Values represent mean ± SD (n = 3). Error bars represent standard deviations based on three independent biological replicates. DNA yields varied non-linearly with palm olein concentration, with peak extraction efficiency observed at 3–10% palm olein. PCR amplification targeting the MT3-B gene yielded a consistent 109 bp product across all samples, confirming successful DNA extraction and amplification regardless of substitution level.

DNA was successfully extracted from all fortified yogurt fat samples. Quantification showed that DNA concentration varied non-linearly with palm olein content, peaking at 3% and 10% palm olein and declining at higher proportions (Fig. 2). This pattern is attributed to matrix effects on DNA recovery and potential PCR inhibitors co-extracted at elevated palm olein levels. To assess reproducibility, DNA concentrations were measured in triplicate and are shown in (Fig. 2) with standard deviation error bars.

#### qPCR analysis and assay validation

Melting curve analysis of qPCR amplicons demonstrated a sharp peak at 83.4 °C, indicating specificity for the MT3-B target. Amplification plots yielded Ct values < 35 for all samples containing ≥ 1% palm olein (Table 1), indicating robust and quantifiable detection. A calibration curve constructed using serial dilutions of palm olein–derived DNA showed strong linearity (R^2^ = 0.999) with amplification efficiency of 97.6%. Detection limits determined using DNA standards were 0.01 ng for the limit of detection (LOD) and 0.02 ng for the limit of quantification (LOQ), satisfying typical food authenticity detection requirements.

**Table 1:** Quantitative PCR (qPCR) cycle threshold (Ct) values for serial dilutions of palm olein DNA using the MT3-B marker (mean ± SD, n = 3).

Sample ID	DNA Input (ng)	Ct Value (Mean ± SD)
100	20.00	26.91 ± 0.11
50	10.00	27.64 ± 0.30
30	6.00	28.37 ± 0.30
10	2.00	30.16 ± 0.71
5	1.00	31.46 ± 0.34
3	0.75	32.56 ± 0.39
1	0.25	35.77 ± 0.04
0.1	0.02	38.56 ± 0.19
0.05	0.01	39.80 ± 0.34
NTC	0.00	No amplification

DNA input reflects total ng of palm olein genomic DNA per qPCR reaction. NTC: No-template control. The limit of detection (LOD) was established at 0.01 ng DNA, and the limit of quantification (LOQ) at 0.02 ng. qPCR was performed using MT3-B primers with SYBR Green chemistry. See Methods section for thermocycling profile.

### GC-FID analysis of phytosterols

**Fig. 3:**
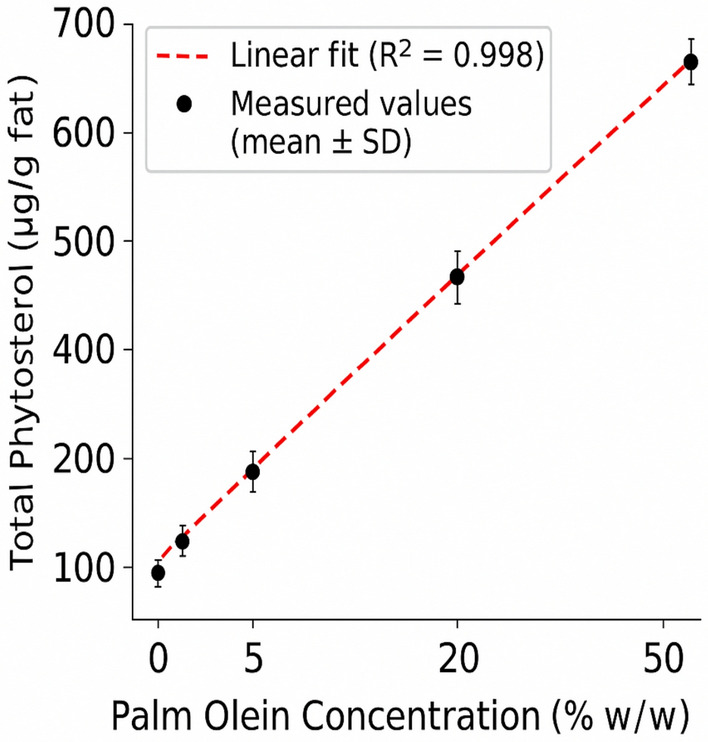
Calibration curve for total phytosterols content versus palm olein concentration. Data points represent mean phytosterols concentrations from triplicate fortified yogurt samples (0.1–30% w/w palm olein). A linear regression was fitted (R^2^ = 0.998), and shaded area represents the 95% confidence interval. This calibration was used to quantify phytosterols in commercial and model samples.

GC-FID chromatograms displayed a progressive, nearly exponential rise in total phytosterol content with increasing palm oil inclusion (Fig. 3). Differences were most pronounced at ≥ 30% palm oil substitution. Total phytosterol levels ranged from 0.08% in authentic yogurt fat to 1.4% at 50% palm-oil substitution. Calibration curves for individual sterols showed R^2^ ≥ 0.998 and LODs between 0.05–0.1% w/w.

A calibration curve for β-sitosterol was constructed using eight concentration points (1–50% palm olein w/w), plotted as individual data points with linear regression (Fig. 3). This range reflects plausible levels of partial substitution or additive use in dairy products. A 100% palm olein sample was included only as a qualitative reference to illustrate the sterol profile of the pure adulterant and was excluded from the regression model. The regression model demonstrated excellent linearity with R^2^ = 0.998. The limit of detection (LOD) and limit of quantification (LOQ), calculated based on standard deviation of response and slope (as per ICH Q2(R1)), were 0.05% and 0.10% palm olein w/w, respectively.

To evaluate the applicability of the dual-method strategy in real-world settings, we analysed 15 commercially available yogurt samples. A detailed summary of each sample is provided in Supplementary Table S1, including product label descriptions, nutritional information (e.g., fat content), MT3-B qPCR Ct values, and phytosterol concentrations (µg/g fat). Several products showed detectable levels of β-sitosterol and campesterol despite not declaring any vegetable oils or emulsifiers on the label, suggesting possible undeclared use of palm-derived additives.

All 15 commercial yogurt samples tested positive for palm-derived markers using both qPCR and GC-FID. Phytosterol concentrations ranged from 412.5 to 1031.2 µg/g fat, with β-sitosterol and campesterol consistently detected above baseline levels observed in authentic dairy fat. Corresponding qPCR Ct values for MT3-B gene amplification ranged from 33.7 to 35.7, indicating low but detectable levels of palm DNA. Supplementary Table S1 provides detailed data for each sample, including quantified sterol levels, MT3-B Ct values, labelled fat content, and presence of declared vegetable oils or emulsifiers. These results support the interpretation that palm-based components—either as additives or undeclared oils—are present in the tested products.

**Fig. 4:**
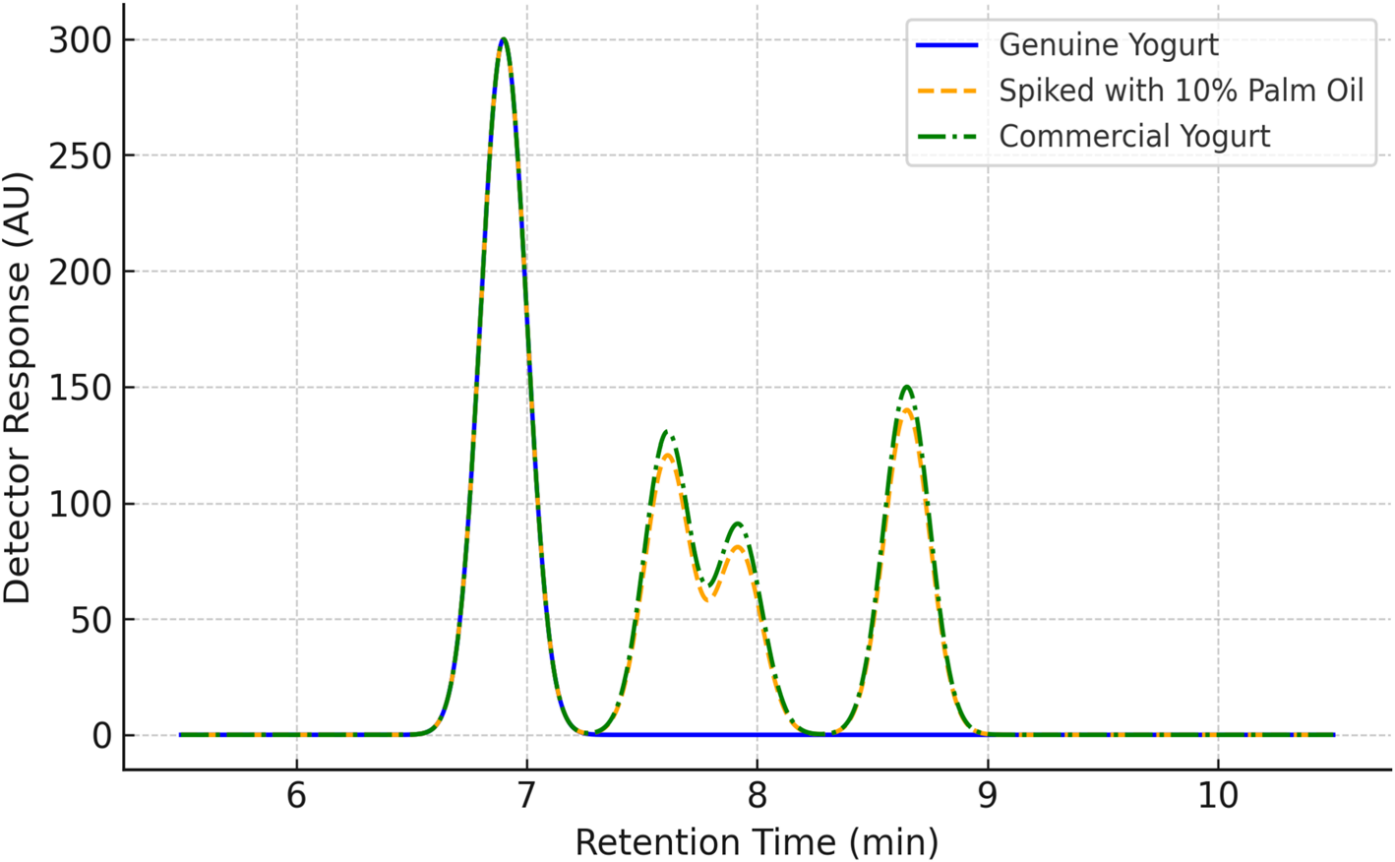
Overlaid GC-FID chromatograms of yogurt lipid extracts. Chromatograms are shown for: (i) a genuine dairy yogurt sample (blue), (ii) a yogurt sample spiked with 10% palm olein (orange), and (iii) a representative commercial yogurt sample (red). Peaks correspond to cholesterol (CHOL), campesterol (CAM), stigmasterol (STIG), β-sitosterol (B-SIT), and internal standard (betulin, IS). Clear differences in phytosterol peak intensity confirm the detection of palm-derived plant sterols in adulterated and commercial products.

A representative GC-FID chromatogram (Fig. 4) displayed well-resolved peaks corresponding to cholesterol and characteristic palm-derived phytosterols (β-sitosterol, campesterol, and stigmasterol). Peak assignments and retention times are summarized in (Table 2). Betulin was used as an internal standard, and its retention time (~ 10.8 min) is now labelled in the chromatogram.

**Table 2:** Phytosterol content in fortified and commercial yogurt fat samples determined by GC-FID (mean ± SD, n = 3).

**Sample ID**	**Palm olein (%)**	**β-Sitosterol (μg/g fat)**	**Campesterol (μg/g fat)**	**Stigmasterol (μg/g fat)**	**Total phytosterols (μg/g fat)**
F0	0%	ND	ND	ND	ND
F1	1%	10.3 ± 1.1	4.8 ± 0.6	2.1 ± 0.3	17.2 ± 1.5
F2	3%	28.1 ± 2.3	12.9 ± 1.1	4.8 ± 0.5	45.8 ± 3.4
F3	5%	46.7 ± 3.0	20.2 ± 1.4	7.6 ± 0.8	74.5 ± 4.2
F4	10%	89.4 ± 4.5	39.1 ± 2.5	13.7 ± 1.2	142.2 ± 5.6
F5	30%	260.3 ± 9.6	112.5 ± 6.3	38.6 ± 2.1	411.4 ± 10.7
F6	50%	421.8 ± 12.2	181.3 ± 7.2	60.2 ± 3.3	663.3 ± 12.8
F7	100%	832.5 ± 15.7	363.4 ± 9.5	119.7 ± 4.6	1315.6 ± 18.1
C1	—	68.2 ± 3.5	32.4 ± 2.2	11.5 ± 0.9	112.1 ± 4.1
C2	—	74.6 ± 4.1	36.7 ± 1.9	13.9 ± 1.0	125.2 ± 4.3
C3	—	95.1 ± 5.2	41.3 ± 2.5	15.8 ± 1.1	152.2 ± 5.8
C4	—	81.7 ± 4.4	38.9 ± 2.3	14.3 ± 0.9	134.9 ± 4.9
C5	—	63.9 ± 3.2	27.8 ± 1.6	10.7 ± 0.8	102.4 ± 3.7

F0–F7: Fortified model yogurt fat samples with increasing proportions of palm olein. C1–C5: Commercial yogurt fat samples with no declared palm oil content. ND: Not detected (below LOD of 0.05 μg/g fat). Total phytosterols = sum of β-sitosterol, campesterol, and stigmasterol. All values corrected using betulin as internal standard and external calibration curves (R^2^ > 0.998).

To illustrate the chromatographic differentiation between authentic and adulterated samples, Fig. 4 presents overlaid GC-FID chromatograms from (i) a genuine yogurt sample containing only milk fat, (ii) a sample fortified with 10% palm olein, and (iii) a representative commercial yogurt product. Peaks corresponding to cholesterol, β-sitosterol, campesterol, and stigmasterol are labeled for comparison. As expected, the adulterated and commercial samples exhibited prominent phytosterol peaks absent or negligible in the authentic sample, supporting the specificity of the method. Complete phytosterol quantification data for all spiked and commercial samples are provided in Supplementary Table S1, with sterol concentrations expressed in µg/g fat and normalized to the internal standard (betulin).

### Method comparison and correlation

A comparative assessment of the MT3-B qPCR assay and GC-FID sterol profiling is presented in Table 3. While qPCR offered rapid and highly specific detection of DNA from oil palm (Elaeis guineensis), GC-FID enabled precise quantification of phytosterols. A strong positive correlation (r = 0.89) between DNA concentration (Ct values) and total phytosterol content affirmed the complementary nature of these methods.

**Table 3:** Comparison of qPCR and GC-FID methods for detecting palm olein adulteration in yogurt.

Parameter	qPCR (MT3-B gene detection)	GC-FID (Phytosterol profiling)
Estimated time per sample	2–3 h	4–6 h
Estimated reagent cost per sample	$3–5	$10–15
Required instrumentation	Real-time PCR thermal cycler	Gas chromatograph with FID
Internal standard used	Not applicable	Betulin
Typical sample throughput	Up to 96 samples (plate format)	10–15 samples (autosampler batch)
Method sensitivity	Detects target DNA from trace sources	Detects sterol markers above LOQ
Key limitations	Requires specific primers, DNA purity	Sample prep and derivatization required
Sample preparation complexity	Moderate	High

LOQ = Limit of quantification. GC-FID = Gas Chromatography–Flame Ionization Detection. qPCR = quantitative polymerase chain reaction. All time and cost estimates refer to individual sample processing under standard laboratory conditions. Time values represent active hands-on and runtime per sample. qPCR timing is based on 96-well plate batch processing but normalized per sample. Cost estimates include reagents and consumables only and do not account for technician labour, equipment depreciation, or institutional overhead. These assumptions are further detailed in Supplementary Table S2.

Cost and time were estimated based on reagent and consumable usage, average workflow duration, and method-specific throughput. ‘Time per assay’ refers to the average processing time per individual sample rather than per batch. For qPCR, while reactions are typically run in a 96-well format, the reported time and cost values reflect a per-sample basis. Technician labour and instrument depreciation were excluded from cost estimates to maintain consistency across methods and focus on direct consumables. Table 3 compares the performance characteristics of the qPCR and GC-FID methods used in this study. To enhance transparency, approximate per-sample costs and processing times have been included based on routine laboratory practice. The estimated cost of qPCR (including primers, SYBR Green master mix, and consumables) is $3–5 per sample, with a typical turnaround time of 2–3 h. GC-FID analysis, which involves derivatization reagents, helium gas, and wear on chromatographic columns, is estimated at $10–15 per sample with a run time of 3–4 h. These values reflect reagent and equipment use, but not labour or instrument amortization.

qPCR offered faster turnaround and higher sensitivity, while GC-FID provided quantitative sterol profiles with complementary specificity. The strong positive correlation between DNA Ct and phytosterol content (r = 0.89) supports the complementary utility of these methods.

### Application to commercial samples

To evaluate the real-world performance of the combined molecular and chromatographic detection methods, 15 high-fat yogurt samples from well-established commercial brands were analysed. These products were marketed as dairy-based with no indication of plant oil supplementation on their ingredient labels. Analysis of 15 commercial yogurt samples revealed trace palm-derived DNA (Ct 33.2–35.7) and total phytosterol concentrations between 0.87–1.40% of total fat. These findings indicate that all products contained undeclared palm-derived components despite label claims of 100% dairy fat. The convergence of molecular and chromatographic evidence underscores the effectiveness of the combined method for real-world authenticity surveillance and highlights the need for multi-analytical testing in regulatory compliance.

DNA extraction followed by qPCR targeting the MT3-B gene resulted in amplification across all 15 samples, with cycle threshold (Ct) values ranging from 33.2 to 35.7. These Ct values fall within the detection range established in the laboratory-spiked yogurt matrix, indicating the presence of trace amounts of palm-derived DNA.

Complementary GC-FID analysis revealed quantifiable levels of plant sterols in all 15 samples, with total phytosterol concentrations ranging from 0.87% to 1.4% of total fat. The sterol profiles were consistent with the presence of β-sitosterol and campesterol, which are characteristic markers of palm oil adulteration.

These results demonstrate that combined molecular and chromatographic detection strategy.is effective in identifying low-level palm oil adulteration in commercial yogurt products. The detection of both molecular and chemical indicators in products not labelled for plant oil inclusion underscores the importance of multi-analytical surveillance in supporting food authenticity and consumer protection.

## Discussion

Detecting palm oil adulteration in dairy matrices demands analytical methods that balance sensitivity, throughput, and adaptability. GC-FID and GC–MS remain the industry benchmarks for the quantification of phytosterols and can clearly detect plant oil markers such as β-sitosterol and campesterol^8^. However, the labour-intensive nature of gas chromatographic workflows limits their scalability for routine quality control, especially in decentralized or resource-limited environments^6^. DNA-based molecular assays, such as qPCR targeting the oil palm–specific MT3-B gene, offer a complementary highly specific route for rapid adulteration screening^10^. Combination molecular and chromatographic testing —as recommended by food authenticity experts—is a layered strategy to enhance the accuracy and confidence of food fraud surveillance^11^. For complex dairy matrices, particularly those with high protein and fat content—alternative rapid-screening tools like near-infrared spectroscopy coupled with chemometric modelling have also proven effective for distinguishing authentic versus adulterated samples with minimal sample preparation^12^.

In the present study, the MT3-B qPCR assay demonstrated robust performance, achieving a limit of detection (LOD) of 0.05% (w/w), high amplification efficiency (~ 98%), and strong linearity (R^2^ ≥ 0.999), with intra- and inter-assay CVs < 10% across replicates. This qPCR assay provides a practical, rapid first-line detection tool. The complementary GC-FID method yielded quantitative sterol profiles with comparable sensitivity (LOD 0.05% w/w) and excellent calibration curve fit (R^2^ ≥ 0.998). Together, these two analytical tools enhance both the reliability and traceability of palm olein detection workflows for dairy product authenticity. The MT3-B qPCR assay demonstrated a detection limit of 0.05% (w/w), which exceeds the sensitivity required to detect unauthorized fat substitution under international guidelines. For instance, the European Union threshold for milk fat substitution is 1% w/w (EU Regulation 273/2008), while Codex Alimentarius standards specify that milk fat must be the sole source of fat in standardized dairy products. Our LOD therefore falls well below these regulatory thresholds, demonstrating the method’s suitability for compliance monitoring.

Application of this dual-analytical strategy to 15 commercial yogurt samples revealed trace palm-derived components in all tested products (Ct 33.2–35.7; total phytosterols 0.87–1.40% w/w), despite labels indicating 100% dairy fat. This underscores the practical utility of the combined approach for real-world authenticity testing and supports its incorporation into routine market surveillance, especially in cases where labelling is incomplete or misleading.

The relevance of detecting vegetable oil adulterants in dairy is well established. Previous investigations have shown the substitution of milk fat with palm, soybean, or canola oil in liquid milk and cheese products^5,9,13^. These practices distort the nutritional profile and may mislead consumers. Our study extends this scrutiny to yogurt, a matrix where fat authentication is more challenging due to emulsification and processing.

The detection of β-sitosterol and campesterol alone is not sufficient to conclusively confirm palm oil adulteration, as these sterols are common to multiple vegetable oils and may also be introduced via trace contaminants or permitted additives. However, in our study, their concentrations in commercial samples were significantly elevated compared to the authentic control. When interpreted alongside the presence of the MT3-B chloroplast DNA marker—specific to Elaeis guineensis—the likelihood of palm-derived fat inclusion is greatly strengthened. The combined molecular and chemical evidence therefore enhances detection specificity and supports a more confident interpretation of potential adulteration.

Combining molecular and chromatographic analyses offers independent and complementary evidence for detecting palm oil adulteration, making this strategy especially useful in complex dairy products like yogurt. qPCR targeting the MT3-B gene offers high sensitivity and species specificity but may be limited by DNA degradation in processed products. GC-FID sterol profiling complements this by quantifying chemical markers that are thermally stable and structurally diagnostic. When used together, these methods enhance detection robustness in both laboratory and field-adaptable settings, improving confidence in authenticity assessments where reliance on a single marker could lead to false negatives or ambiguous results.

While our findings demonstrate the utility of combining MT3-B qPCR and GC-FID sterol profiling for detecting palm-derived adulteration in yogurt, several limitations must be acknowledged. First, the detection of oil palm DNA in commercial samples does not confirm deliberate substitution, as palm-based additives (e.g., emulsifiers, mono- and diglycerides) may also carry trace DNA. Moreover, refined palm oils often undergo processing steps that degrade nucleic acids, potentially leading to false negatives or underestimation of DNA presence. Similarly, although β-sitosterol and campesterol are reliable indicators of plant oil inclusion, they are not unique to palm oil and may occur in other vegetable-derived ingredients. Therefore, sterol detection alone cannot specify adulterant origin without supporting molecular evidence. In this context, the dual-method approach enhances specificity, but should still be interpreted with caution in highly processed matrices.

While our spiked model samples allowed for controlled calibration of both qPCR and GC-FID assays, we did not directly assess extraction efficiency for palm olein components from yogurt matrices. This represents a limitation of the current study. Future work should incorporate formal spike-recovery experiments to quantify the recovery rates of DNA and phytosterols from emulsified dairy systems. Such data would strengthen the reliability of molecular and chemical detection in real-world authenticity assessments. While qPCR and GC-FID are individually established techniques in food authentication, this study offers three additional contributions beyond their combined application. First, we optimized a DNA extraction protocol tailored for emulsified, lipid-rich dairy matrices—preserving template quality for reliable MT3-B gene amplification. Second, our GC-FID method was fully validated in a yogurt matrix using certified standards, internal normalization (betulin), and detailed calibration parameters (R^2^, LOD, LOQ). Third, we demonstrated the real-world utility of this dual approach by screening 15 commercial yogurt products, all of which showed evidence of undeclared palm oil components. The corresponding DNA and sterol data are provided in Supplementary Table S1 to support transparency and reproducibility.

While 15 commercial yogurt samples tested positive for palm oil DNA and phytosterols, these products were selected without prior knowledge of composition and may contain palm-derived components such as emulsifiers or monoacylglycerols rather than direct milk fat substitution. A single authentic control yogurt made solely from dairy fat was used as a negative reference. Although clear differences were observed in MT3-B gene amplification and sterol profiles, we acknowledge that broader profiling of verified palm-free yogurts would improve confidence in marker specificity and range. Future studies should include a larger panel of certified authentic dairy products to refine detection thresholds and differentiate between minor additive inclusion and economic adulteration. Because the commercial yogurt samples were analysed as purchased, without any modification, the specific origin of the palm-derived components could not be verified. The presence of palm DNA or sterols may reflect either undeclared substitution of milk fat or the inclusion of palm-based additives such as emulsifiers or monoacylglycerols. Without detailed formulation data, distinguishing between adulteration and legitimate additive use is not possible. To support transparency in interpretation, Supplementary Table S1 now provides label information for each commercial product, including declared fat content, presence of vegetable oils, and use of additives.

It is well established that refining processes such as bleaching and high-temperature steam distillation (> 200 °C) can degrade or eliminate detectable DNA in edible oils. Therefore, the detection of the MT3-B gene in commercial yogurt samples does not necessarily imply the direct inclusion of fully refined palm oil. A more plausible explanation is the presence of palm-derived ingredients such as mono- and diglycerides, palm stearin, or emulsifiers that retain trace amounts of chloroplast DNA due to partial processing. We explicitly acknowledge that the exact source of the DNA signal in these samples cannot be definitively determined from the current data. This limitation underscores the need for targeted spiking studies involving known palm-based additives to clarify DNA persistence and specificity in complex food matrices.

To aid interpretation of molecular and sterol detection results, we expanded Supplementary Table S1 to include labelling information for all commercial yogurt products. This includes total fat content (g/100 g), declared use of vegetable oils or emulsifiers, and any statements such as ‘made from 100% milk.’ Notably, several products containing detectable palm markers did not disclose the use of non-dairy fats on their ingredient labels. This discrepancy underscores the value of analytical surveillance to identify undeclared additives and highlights the potential for consumer misinformation.

This study focused on the detection of palm oil adulteration using an MT3-B gene-specific qPCR assay and GC-FID-based sterol profiling. The molecular method is specific to Elaeis guineensis and does not detect DNA from other plant oil sources. However, the GC-FID approach has broader applicability, as different vegetable oils exhibit characteristic sterol fingerprints. For instance, high levels of brassicasterol and altered β-sitosterol/campesterol ratios are indicative of canola oil, while soybean oil is often marked by elevated stigmasterol. Although the detection of non-palm oils was beyond the scope of this work, the analytical framework presented here can be adapted to other adulterants by selecting suitable molecular markers and establishing sterol-based calibration models for alternate oil types. It is important to note that MT3-B qPCR and GC-FID represent fundamentally different analytical approaches: the former detects species-specific DNA from oil palm (Elaeis guineensis), while the latter quantifies plant-derived sterol compounds. These methods are not directly comparable in terms of target or signal output. Instead, their complementarity lies in providing orthogonal evidence—molecular and chemical—that, when used together, increases confidence in detecting palm-derived ingredients in complex or processed dairy matrices.

Although this study was designed around yogurt as a representative emulsified dairy matrix, the combined molecular and chemical detection strategy may be adaptable to other dairy products, such as processed cheese or milk powder. However, each matrix presents unique analytical challenges—such as DNA degradation during spray drying or protein-fat interactions during fermentation—that must be addressed through matrix-specific validation. Importantly, we emphasize that qPCR-based detection of palm DNA should not be interpreted in isolation. Due to the possibility of trace contamination or additive presence, Molecular results should be supported by confirmatory chemical markers, such as phytosterol profiling via GC-FID, to ensure reliable identification and authentication. In such contexts, chemical approaches offer a complementary confirmatory method that can verify adulteration when molecular detection is limited by DNA degradation or matrix complexity. It is important to note that MT3-B qPCR and GC-FID represent fundamentally different analytical approaches: the former detects species-specific DNA from oil palm (Elaeis guineensis), while the latter quantifies plant-derived sterol compounds. These methods are not directly comparable in terms of target or signal output. Instead, their complementarity lies in providing orthogonal evidence—molecular and chemical—that, when used together, increases confidence in detecting palm-derived ingredients in complex or processed dairy matrices.

While molecular assays provide rapidity and specificity, DNA recovery can be limited by fat-rich matrices, and inhibitors can compromise qPCR efficiency if not adequately removed during extraction^12^. Chemical methods offer a complementary, quantitative confirmation of adulterants; however, they cannot by themselves confirm the source of the plant oil in mixed-adulterant scenarios or discriminate palm oil derivatives used as processing aids. Real-world cases of dairy fraud reported across global supply chains further highlight the need for robust multi-analytical confirmation strategies^14^. Our study was limited to yogurt, and further validation will be required before extending this method to other dairy products such as milk powder or cheese. Matrix-dependent factors like lipid content, emulsification state, and thermal history can all affect DNA recovery and sterol extraction. Additional studies using larger and more diverse sample sets—including known negatives and spiked additives—are needed to establish detection thresholds and improve robustness.

Finally, while the results from 15 commercial samples suggest the presence of undeclared palm-based ingredients, we cannot definitively determine the source or intent of inclusion. Regulatory interpretations should therefore be informed by both analytical results and comprehensive labelling assessments. Emerging tools, including nano-biosensors and machine-learning-enhanced chemometric techniques, show promise for real-time, portable, and field-deployable adulterant screening in complex food matrices^15,16^. Multiplex PCR and compact spectroscopic platforms could further extend detection capabilities to a broad range of potential adulterants beyond palm oil, enabling a more scalable and adaptive solution for evolving food fraud risks^16,17^. Integrating these innovative approaches into multi-analytical protocols will be vital to improving food authenticity assurance and protecting consumers across global dairy industries.

## Methods

### Sample preparation

Yogurt used for palm olein fortification experiments was obtained from a reputable local dairy, selected to ensure the absence of palm oil during its manufacture. Edible palm olein was sourced from the local market. Authentic control yogurt (n = 1) was sourced from a local dairy known to use no plant-derived additives.

For lipid extraction, 250 g of yogurt was weighed, homogenized, and transferred to an Erlenmeyer flask. Lipids were extracted following the AOAC official method (Horwitz and Latimer, 1975) using a solvent mixture (Solvent A) comprising equal volumes of methanol, hexane, chloroform, petroleum ether, and benzene. The mixture was allowed to stand in a dark environment for 48 h before filtration through filter paper. The filtrate was evaporated at 50 °C to remove residual solvents, followed by additional drying at 105 °C for 30 min to eliminate moisture.

Model yogurt fat samples spiked with known concentrations of palm olein (ranging from 1 to 100%, w/w) were prepared to establish calibration curves and validate the analytical methods. These fortified samples served as controlled standards for evaluating assay sensitivity and linearity. In contrast, the 15 commercial yogurt products were analysed in their original form, without any added palm oil or modification.

The fat extracted from plain full-fat yogurt (without palm oil) was melted at 50 °C and used as the base lipid matrix. This matrix was spiked with palm olein oil at defined proportions to obtain fortified samples with palm olein concentrations of 0%, 1%, 3%, 5%, 10%, 30%, 50%, and 100% (w/w). These blends were homogenized thoroughly and used for molecular and chromatographic analyses.

Prior to UV/vis spectrophotometric analysis, lipid extracts were diluted in hexane to ensure absorbance readings fell within the linear range of 0.2–0.8 optical density (OD), minimizing potential signal distortion due to concentration effects.

### Detection of _MT3-B_ gene by PCR

#### DNA extraction

DNA extraction was performed using a modified CTAB method, where 10 mL of lipid sample was mixed with 2 mL of CTAB buffer, 2 mL hexane, and 200 µL Tween 80. The mixture was incubated at 65 °C for 60 min with intermittent inversion, followed by chloroform-isoamyl alcohol extraction and isopropanol precipitation at − 20 °C. The DNA pellet was washed with 1 mL of 70% ethanol, followed by air-drying in a laminar flow hood. The resulting DNA was resuspended in 100 µL TE buffer (10 mM Tris–HCl, 1 mM EDTA, pH 8.0).DNA concentration and purity were measured using a Nanodrop 2000 spectrophotometer (Thermo Scientific, USA). DNA concentration was calculated as shown in Eqn. (1):

1 $${\text{DNA concentration }}\left( {{\text{ng}}/\mu {\text{L}}} \right)\, = \,{5}0\, \times \,{\text{Absorbance at 26}}0{\text{ nm}}$$

All spectrophotometric DNA quantification measurements were conducted in biological triplicate (n = 3). Reported values represent the mean ± standard deviation, and error bars in Fig. 2 reflect this variability.

#### PCR Amplification using _MT3-B_ gene primers

The following primers were used to amplify the MT3-B chloroplast gene of Elaeis guineensis, as reported by Zhang et al. (2009): forward 5-TCCAGGAGATGTGGAGCAAG-3 and reverse 5-CCTTGTAGCCGACCTTGAAG-3. These primers target a 109 bp region specific to palm chloroplast DNA and have demonstrated high specificity and amplification efficiency^18^.

Conventional PCR was carried out using a PeQlab Thermo Cycler 96Grad with primers targeting the MT3-B gene, as previously described by Zhang et al. (2009). Each 20 µL reaction mixture contained 10 µL of 2 × master mix (0.2 U/µL Taq DNA polymerase, 3 mM MgCl₂, 0.4 mM dNTPs, and 0.2% Tween 20 in Tris–HCl buffer, pH 8.5), 50–100 ng of template DNA, and 0.5 µM of each forward and reverse primer. Thermal cycling was initiated with denaturation at 94 °C for 5 min, followed by 40 cycles of 20 s at 94 °C, 30 s at 58 °C for primer annealing, and 20 s at 72 °C for extension. A final extension was carried out at 72 °C for 5 min^18^.

#### Gel electrophoresis and quantitative PCR

PCR products (8 µL) were resolved on a 2% agarose gel in TBE buffer at 120 V for 50 min. Bands were visualized using a CCD-5 gel documentation system (Qiagen).

Quantitative PCR (qPCR) was performed using a StepOne real-time PCR system (Applied Biosystems, USA) and SYBR Green 2 × Master Mix (Ampliqon, Denmark) Reactions (10 µL) contained 5 µL SYBR Green Mix, 0.25 µL of each primer, and 1 µL DNA template and nuclease-free water.

Quantitative PCR was performed using a StepOne Real-Time PCR system with an initial denaturation at 95 °C for 15 min, followed by 40 amplification cycles comprising 15 s at 94 °C, 30 s at 58 °C for primer annealing, and 20 s at 72 °C for extension. A melting curve analysis was conducted from 65 °C to 95 °C in 0.3 °C increments to confirm amplification specificity.

### Gas chromatographic analysis of phytosterols

Lipid blends were extracted using a modified Folch method and saponified with ethanolic KOH to release free sterols. The unsaponifiable fraction was isolated with hexane and derivatized using *N, O*-bis (trimethylsilyl) trifluoroacetamide (BSTFA) containing 1% trimethylchlorosilane (TMCS) to produce trimethylsilyl ethers.

Gas chromatography was performed on a GC-FID system (Agilent 7890) equipped with a DB-5MS capillary column (30 m × 0.25 mm i.d., 0.25 μm film thickness). The injection volume was 1 μL in splitless mode. The oven temperature program started at 250 °C (held for 1 min), increased to 300 °C at 10 °C/min, and held for 10 min, yielding a total runtime of approximately 28 min. The retention times of target analytes were: cholesterol (18.4 min), campesterol (20.6 min), stigmasterol (22.1 min), β-sitosterol (23.4 min), and betulin (internal standard, 25.0 min). FID detector temperature was maintained at 320 °C.

These conditions provided baseline separation of β-sitosterol, campesterol, and stigmasterol, with retention time differences > 0.5 min and resolution (R) > 1.5 in all cases. Identity confirmation was performed using authenticated standards, and reproducibility was assessed across triplicate injections, yielding RSD values below 5%.

Betulin was used as an internal standard and added at a fixed concentration prior to derivatization. It eluted at approximately 6.5 min and was used to normalize sterol quantification across all samples. Sterols were quantified using external calibration curves prepared from certified standards of β-sitosterol, campesterol, and stigmasterol (Sigma-Aldrich, ≥ 95% purity). Calibration was performed over the range 0.5–50 µg/mL, with each point run in triplicate. Quantitative values were calculated by normalizing the peak area of each sterol to that of the internal standard (betulin) and converting to μg/g of fat. Calibration curves for β-sitosterol demonstrated excellent linearity (R^2^ = 0.998), with detection and quantification limits determined to be 0.05% and 0.10% (w/w), respectively. All reported concentrations in Table 2 reflect mean values from three independent biological replicates. 

### Validation and statistical analysis

Quantitative data were analysed in Microsoft Excel. qPCR cycle threshold (Ct) values were used to calculate LODs and LOQs. All measurements were conducted in triplicate to ensure precision and reproducibility.

Validation parameters for the GC-FID method were calculated according to ICH Q2 (R1) and AOAC guidelines. The linearity of β-sitosterol response was assessed over a range of 0.1–30% palm olein (w/w), and LOD and LOQ were estimated from the standard deviation of the response (σ) and slope (S) using the equations LOD = 3.3(σ/S) and LOQ = 10(σ/S).

Method validation followed established analytical chemistry guidelines (ICH Q2 (R1); AOAC 2016). Linearity was evaluated by constructing calibration curves for β-sitosterol over a concentration range of 1–50% palm olein (w/w), and linear regression was applied to assess correlation (R^2^). The limit of detection (LOD) and limit of quantification (LOQ) were calculated using the standard deviation of the response (σ) and the slope of the calibration curve (S) according to the formulas: LOD = 3.3(σ/S) and LOQ = 10(σ/S). Precision was assessed by triplicate measurements of fortified samples across the concentration range, with results expressed as relative standard deviation (RSD). All sterol quantification results reported RSD values below 5%, indicating acceptable reproducibility.

## Supplementary Information

Supplementary Information 1.

Supplementary Information 2.

## Data Availability

All data generated or analysed during this study are included in this published article and its supplementary information files. Supplementary Table S1 presents detailed phytosterol profiling and qPCR data for all fortified and commercial yogurt samples, including concentrations of β-sitosterol, campesterol, and stigmasterol (µg/g fat), total phytosterol content, MT3-B qPCR cycle threshold (Ct) values, declared fat content, and product labelling information. All sterol concentrations are normalised to internal standard and fat content. Supplementary Table S2 provides the assumptions and calculation basis for time and cost estimates reported in Table 3, including reagent requirements, processing steps, per-sample runtime, and estimated costs for both qPCR and GC-FID methods.
