# Supplementary material for: Comparative analysis of molecular and chromatographic methods for detecting palm oil adulteration in yogurt
Source: Sci Rep. 2025 Aug 12;15:29568. doi: 10.1038/s41598-025-15523-6 (PMC12343945; doi:10.1038/s41598-025-15523-6)
Supplement: Supplementary file 1 — Supplementary Information 1. [file 41598_2025_15523_MOESM1_ESM.docx]

**Comparative analysis of molecular and chromatographic methods for detecting palm oil adulteration in yogurt**

**Mohammad Dowlatabadi¹, Seyed Ali Mortazavi²*, Hasan Ravansalar³, Mohammad Reza Saedi Asl¹, Ahmad Pedramnia¹**

¹ Department of Food Science and Technology, Sab.C., Islamic Azad University, Sabzevar, Iran
² Department of Food Science and Technology, Faculty of Agriculture, Ferdowsi University of Mashhad, Mashhad, Iran
³ Department of Microbiology, Faculty of Medicine, Sabzevar University of Medical Science, Sabzevar, Iran

[^*^mortazaviali802@gmail.com](mailto:*mortazaviali802@gmail.com)

**Table S1. Phytosterol Profiles and MT3-B qPCR Results for Fortified and Commercial Yogurt Samples**

This table includes: (i) concentrations of β-sitosterol, campesterol, and stigmasterol (µg/g fat), (ii) total sterols, (iii) Ct values from qPCR detection of the MT3-B gene, (iv) declared fat content, and (v) product labeling information (e.g., ‘vegetable oils,’ emulsifiers). All sterol concentrations are normalized to internal standard and fat content. Data are provided for all 15 fortified and 15 commercial yogurt samples.

| **Sample ID** | **Brand/Label Description** | **Fat Content (g/100g)** | **Declared Additives (e.g., E471, emulsifiers)** | **Vegetable Oils Declared** | **“100% Milk” Claim** | **MT3-B Ct (Mean ± SD)** | **β-Sitosterol (μg/g fat)** | **Campesterol (μg/g fat)** | **Stigmasterol (μg/g fat)** | **Undeclared Palm Marker Present** |
| --- | --- | --- | --- | --- | --- | --- | --- | --- | --- | --- |
| YG-C01 | Full-fat yogurt, local brand | 3.2 | E471, mono-/diglycerides | No | Yes | 34.9 ± 0.28 | 106.4 | 41.7 | 29.3 | Yes |
| YG-C02 | Creamy yogurt, multinational brand | 3.5 | Stabilizers (E1442), emulsifier | No | No | 33.2 ± 0.32 | 92.8 | 36.1 | 24.9 | Yes |
| YG-C03 | Yogurt with fruit base | 3.0 | Fruit puree, thickener | No | No | 35.3 ± 0.25 | 87.5 | 30.4 | 19.6 | Yes |
| YG-C04 | Traditional set yogurt | 3.6 | None declared | No | Yes | 34.7 ± 0.41 | 111.6 | 44.0 | 28.7 | Yes |
| YG-C05 | Low-fat yogurt | 2.0 | Modified starch, E412 | No | No | 35.5 ± 0.30 | 95.3 | 38.1 | 22.4 | Yes |
| YG-C06 | Flavored yogurt with additives | 2.8 | E471, E466, E410 | No | No | 35.7 ± 0.21 | 103.9 | 40.2 | 26.1 | Yes |
| YG-C07 | Whole milk yogurt | 3.8 | E407, natural flavor | No | Yes | 34.2 ± 0.27 | 110.7 | 42.5 | 30.0 | Yes |
| YG-C08 | Fruit yogurt with cereal | 3.1 | E471, cereal extract | No | No | 33.9 ± 0.22 | 101.5 | 39.6 | 27.5 | Yes |
| YG-C09 | Yogurt with no preservatives | 3.5 | Guar gum, pectin | No | Yes | 34.5 ± 0.33 | 98.4 | 37.3 | 25.2 | Yes |
| YG-C10 | Probiotic yogurt | 3.2 | None declared | No | Yes | 33.8 ± 0.26 | 112.9 | 45.1 | 30.7 | Yes |
| YG-C11 | Imported premium yogurt | 4.0 | E471, vegetable stabilizers | Yes | No | 33.4 ± 0.30 | 122.3 | 48.2 | 33.5 | Declared |
| YG-C12 | Drinking yogurt | 2.5 | E466, E471 | No | No | 34.6 ± 0.35 | 91.0 | 35.9 | 21.8 | Yes |
| YG-C13 | Children’s yogurt with vitamin D | 3.4 | Vitamins, E412 | No | No | 35.1 ± 0.29 | 88.1 | 33.6 | 20.5 | Yes |
| YG-C14 | Plain stirred yogurt | 3.6 | None | No | Yes | 34.1 ± 0.27 | 108.5 | 43.2 | 28.0 | Yes |
| YG-C15 | Yogurt with honey | 3.3 | Natural honey, E410 | No | Yes | 35.0 ± 0.28 | 102.7 | 39.8 | 26.3 | Yes |

Notes:

- E471 (mono- and diglycerides) is often palm-derived.
- All products were purchased from supermarkets and stored refrigerated before analysis.
- Ct values >33 indicate low-level detection; all values below 36 considered positive.
- Sterol profiles were obtained from triplicate GC-FID measurements and normalized to μg/g total fat.
- “Undeclared Palm Marker Present” = positive MT3-B signal and/or sterol levels consistent with palm oil (>80 μg/g fat for β-sitosterol based on fortified sample benchmarks).
