# Supplementary material for: Comparative analysis of molecular and chromatographic methods for detecting palm oil adulteration in yogurt
Source: Sci Rep. 2025 Aug 12;15:29568. doi: 10.1038/s41598-025-15523-6 (PMC12343945; doi:10.1038/s41598-025-15523-6)
Supplement: Supplementary file 2 — Supplementary Information 2. [file 41598_2025_15523_MOESM2_ESM.docx]

**Comparative analysis of molecular and chromatographic methods for detecting palm oil adulteration in yogurt**

**Mohammad Dowlatabadi¹, Seyed Ali Mortazavi²*, Hasan Ravansalar³, Mohammad Reza Saedi Asl¹, Ahmad Pedramnia¹**

¹ Department of Food Science and Technology, Sab.C., Islamic Azad University, Sabzevar, Iran
² Department of Food Science and Technology, Faculty of Agriculture, Ferdowsi University of Mashhad, Mashhad, Iran
³ Department of Microbiology, Faculty of Medicine, Sabzevar University of Medical Science, Sabzevar, Iran

[^*^mortazaviali802@gmail.com](mailto:*mortazaviali802@gmail.com)

# **Table S2. Assumptions and calculation basis for time and cost estimates reported in Table 3.**

| **Parameter** | **qPCR (MT3-B gene detection)** | **GC-FID (Phytosterol profiling)** | |
| --- | --- | --- | --- |
| Estimated time per sample | 2–3 hours (normalized from batch 96-well format) | 4–6 hours (including prep, derivatization, and runtime) |  |
| Processing steps included | DNA extraction, qPCR mix setup, thermocycling | Lipid extraction, saponification, derivatization, GC-FID |  |
| Typical sample throughput | Up to 96 samples per plate (batch) | 10–15 samples per autosampler batch |  |
| Per-sample injection time | Not applicable (plate-based) | ~25 minutes |  |
| Injection mode | Not applicable | Splitless |  |
| Injection volume | Not applicable | 1 μL |  |
| Key reagents and consumables | SYBR Green Master Mix, MT3-B primers, PCR tubes, buffers | Saponification reagents, BSTFA + TMCS, solvents, betulin |  |
| Estimated reagent cost per sample | $3–5 | $10–15 |  |
| Instrument requirements | Real-time PCR cycler (96-well compatible) | GC-FID with autosampler |  |
| Internal standard used | Not applicable | Betulin |  |

**Notes:**
All time and cost estimates are based on individual sample processing under typical laboratory conditions. Costs reflect consumables and reagents only, excluding technician labor, equipment depreciation, and overhead. For qPCR, processing time is normalized from parallel processing on a 96-well plate. For GC-FID, time includes sample preparation and chromatographic runtime. Reagents and protocol steps correspond to those described in the Methods section of the main manuscript.
